# A “Crowners ’Quest”

**Published:** 1859-11

**Authors:** 


### A "CROWNER'S 'QUEST."

Our friend, M'Q, of the "Cosmos," has been playing coroner, and has held two inquests over the dead bodies of decayed teeth; and the verdict in each case, is, water in the pulp cavities, in spite of "white wax" administered as a prophylactic.

But lest he should consider this statement not sufficiently *dignified*, for a grave journal, like the "Register," we will be serious.—To demonstrate the correctness of his position, that the pulp cavity will be filled with serum, if not filled with gold, he plugged the cavities of decay, in some extracted teeth, with white wax, and immersed them in water. In several hours, and again, in two days, he and his friends found water in the pulp cavities. We would state that we have repeatedly tried similar experiments, with different results, but they would probably be regarded as *exceptions*, and claimed as proof against us. In a case reported in our September number, the reader will remember that a tooth, with the canal unfilled, remained perfectly healthy, more than eight years, and the canal was still dry. Our friend says of this, it "is one of those instances in which 'the *exception* proves the *rule*'." Of the origin of this phrase we are ignorant. It may contain some latent wisdom; but if it is applied here, according to its true meaning, a more soft and harmless piece of jargon could not well be penned; for, if one exception proves the rule, two afford still stronger proof, and the best established rule is the one that has all the facts arrayed against it. The case referred to flatly contradicts his theory; but he is true to his prototype, of Irish memory, who, when told that his theory was contradicted by *facts*, simply replied, "So much the worse for the facts, then."

In the case reported in our September number, the "confined air" was not "vitiated," nor "most offensive," nor has it been so in similar cases which we have examined. We removed a filling last week (on account of fracture of the tooth) which was inserted ten months ago, and found the canal *dry;* nor could we, or the patient, a sensitive lady, detect any odor, "offensive" or otherwise, emanating from it. But, perhaps this is another *exception,* and it will be claimed as a second witness against us.

But we fully believe that our friend and his jury found water in the pulp cavities. We covered the crown of a molar with wax, and, after due immersion, found water on its grinding surface. Did it first get into the pulp cavity, and pass out through the dentine and enamel? or did it pass between the wax and tooth?

But on the other hand, do those who fill the fangs generally pack the gold so tightly, the whole length of the canal, that there is no space left for the serum to occupy? We know it is sometimes done, for we have done it in favorable cases; but we have examined a goodly number of teeth, for the work in which this degree of perfection was claimed, and found plenty of room for *liquor sanguinis,* or any other liquor.

But our friend asks "in a spirit of inquiry, whether those who are opposed to filling fangs, have not neglected to mention two important objections that have had as much or more weight, in inducing them to assume this position, than anything else, viz: the difficult nature of the operation, and the inadequate compensation they have obtained when performing the task?"

Our answer to this (for ourself only) is an emphatic NO; for we rather like to encounter difficulties, and, if we work for nothing we are proud enough to do as well as we can.

And now, "we would ask in a spirit of inquiry," does our friend, who is so ready to inquire into the motives of others, *feel* that these considerations would be likely to lead him aside from the paths of professional rectitude? Would they tempt him to lay aside his self-respect, and to forget his manhood, and his duties to his fellow man? "We desire to be distinctly understood; these are are not advanced as assertions, but put forward as queries."

But what about filling pulp cavities with plaster of Paris, as we are considerately advised to do *if we don't know how, or can't afford to fill them with gold?* Of course the *liquor sanguinis* will not get into the canal through the *dentinal tubuli,* and the *lacunæ*

and *canaliculi*, if it has plaster in it, or, if it does, it will not likely "spoil." Why not stop out the serum with sponge? It is, perhaps, more porous than plaster. But the question before us is not whether the canal will remain dry or not, but whether or not it should be filled with gold. We began, several years ago, to experiment on the subject, because we thought that fang filling was based on a doubtful theory. We worked our way gradually to our present position. We watched our cases three years before we mentioned the practice to any one, and we have now watched them three years longer, and are satisfied with our success.

And now, reader, this hobby may rest awhile. W.

